# Landscape of national antibiotic utilisation in Malaysia: an analysis of national pharmaceutical sales data from 2019 to 2023

**DOI:** 10.1080/20523211.2026.2668477

**Published:** 2026-05-21

**Authors:** Audrey Huili Lim, Norazida Ab Rahman, Siti Nur Su'Aidah Nasarudin, Abdul Haniff Mohamad Yahaya, Mardhiyah Kamal, Marziaty Shazreena Mohd Shah, Nurul Hidayah Awang, Benedict Lim Heng Sim, Suresh Kumar Chidambaram, Sheamini Sivasampu, Peter Seah Keng Tok

**Affiliations:** a Institute for Clinical Research, National Institutes of Health, Ministry of Health, Shah Alam, Malaysia; b Pharmaceutical Services Programme, Ministry of Health, Petaling Jaya, Malaysia; c Hospital Sungai Buloh, Ministry of Health, Sungai Buloh, Malaysia; d National Public Health Laboratory, Ministry of Health, Sungai Buloh, Malaysia

**Keywords:** Antibiotic utilisation, procurement, DDD, cost, AWI

## Abstract

**Background:**

Evaluating national antibiotic use is crucial for understanding the drivers of antimicrobial consumption and resistance, to develop targeted strategies that promote rational antibiotic use amid limited existing evidence. The aim of this study was to examine the trends and patterns in national antibiotic utilisation and associated expenditure in Malaysia.

**Methods:**

National pharmaceutical sales data from IQVIA was used to obtain data on antibiotics from 2019 to 2023. Antibiotic utilisation rate was presented as defined daily dose (DDD) per 1000 inhabitants per day and expenditure as total cost in Malaysian Ringgit (MYR) per 1000 inhabitants. The proportion of antibiotics was further classified into Access, Watch, Reserve (AWaRe) categories. Compound Annual Growth Rate (CAGR) was used to calculate the changes in utilisation and expenditure rate over time.

**Results:**

Overall, two-thirds of the total antibiotic utilisation was contributed by primary care and the utilisation in the private sector was higher than that in the public sector for both primary care and hospitals. Antibiotic utilisation rate showed a non-significant change in trend between 2019 and 2023 (CAGR = −0.25%; *p* = 0.869). Annual antibiotic expenditure was MYR3525.90 per 1000 inhabitants in 2019 and MYR3822.37 per 1000 inhabitants in 2023, but also exhibited a non-significant trend change (CAGR = 3.1%; *p* = 0.869). The Access category antibiotics accounted for 56.0% of total antibiotic usage, with varying proportions observed between hospitals and clinics and public-private setting. Access-to-Watch Index was 3.18 in public and 1.14 in the private sector. The highest proportion of Watch antibiotics was in private hospitals and the Access antibiotics were highest in public clinics.

**Conclusion:**

While the antibiotic utilisation rate showed a fairly stable trend, the proportion of Access antibiotics falls short of the 60% target set by the WHO. Study findings contribute to informing efforts to strengthen and target stewardship efforts in Malaysia.

## Background

Antibiotics are essential to modern medicine, yet their widespread misuse has led to the rise of resistant pathogens such as ESBL-producing *Enterobacterales* and carbapenem-resistant *Enterobacteriaceae* (CRE), threatening treatment effectiveness (Shaikh et al., 2015; World Health Organisation, 2024). Antimicrobial resistance (AMR) contributed to an estimated 1.14 million deaths in 2021 (Naghavi et al., 2024) and continues to drive up healthcare costs globally (Nelson et al., 2021). According to the World Bank estimates, AMR may lead to US$ 1 trillion additional healthcare costs globally by 2050, and US$ 1–3.4 trillion losses in gross domestic product (GDP) per year by 2030 (World Bank Group). In Malaysia, a crude mortality rate of 9.6% has been reported among patients with multidrug resistant organism infections (WP et al., 2025) while the cost of AMR treatment per admission in Malaysia rose by more than 130% from 2017 to 2019 (AR et al., 2025). This growing crisis underscores the urgent need to understand antibiotic use patterns and their financial and epidemiological impact to inform more effective stewardship and containment strategies.

The surveillance of AMR and antimicrobial consumption or use is among the priorities to address AMR in human health (Ajulo & Awosile, 2024; World Health Organisation, 2023a). As an effort to optimise antibiotic use and kerb AMR, the World Health Organization (WHO) developed the AWaRe classification of antibiotics as a tool to support the monitoring of antibiotic consumption, defining targets and monitoring the effects of stewardship policies (World Health Organisation 2023b). The AWaRe classification system categorises antibiotics into three groups: Access, Watch, and Reserve. The Access group includes first-line antibiotics that are effective, have a lower resistance potential, and should be widely available. The Watch group consists of antibiotics with a higher resistance risk that require careful monitoring and restricted use. The Reserve category comprises last-resort antibiotics reserved for severe multidrug-resistant infections. The AWaRe framework aims to optimise antibiotic use, ensuring that essential antibiotics remain effective while minimising the overuse of high-risk drugs (Saleem, Sheikh, et al., 2025; Sharland et al., 2018; Sharland et al., 2019; Zanichelli et al., 2023). Judicious antimicrobial use is essential to kerb the rise of antibiotic resistance, and this necessitates robust antibiotic stewardship programmes.

In Malaysia, there have been several antibiotic stewardship initiatives by the Ministry of Health such as the National Antibiotic Guidelines, Malaysian Action Plan on Antimicrobial Resistance (MyAP-AMR) and Antimicrobial Stewardship (AMS) Programme (Ministry of Health, 2014, 2019; Ministry of Health (Malaysia), 2024; Ministry of Health and Ministry of Agriculture & Agro-Based Industry Malaysia, 2017) as well as participation in the Global Antimicrobial Resistance and Use Surveillance System (GLASS) (Ministry of Health Malaysia, 2022). A previous study conducted in the Malaysian primary care setting showed that a fair proportion of antibiotics were inappropriately prescribed (Ab Rahman et al., 2016), although improvement in the antibiotic prescribing practice was noted in another study conducted more recently (Lim et al., 2024a). Other works also mainly focused on primary care, including antibiotic utilisation patterns during the COVID-19 pandemic (Lim et al., 2023), as well as a comparison of usage patterns between public and private community healthcare practices (Lim et al., 2024b). Such analyses provide critical insights into the drivers of antibiotic consumption and resistance, enabling policymakers and healthcare professionals to implement targeted strategies to promote rational antibiotic use. Moreover, monitoring national antibiotic use helps track changes over time, assess the influence of public health events, and evaluate the effectiveness of regulatory policies. Thus, this study endeavours to fill this critical gap in knowledge by providing a detailed examination of antibiotic utilisation patterns across healthcare settings in Malaysia. By analysing national antibiotic procurement data from 2019 to 2023, this study aims to examine trends and patterns in antibiotic utilisation and expenditure in Malaysia.

## Methods

### Study design

This is a retrospective study of aggregated pharmaceutical sales records in Malaysia from January 2019 to December 2023.

### Setting

This study was conducted in Malaysia, an upper middle-income country with an estimated population of over 33 million in 2023 (Department of Statistics Malaysia, 2025b). The Malaysian healthcare system comprises the government-led public sector and the fee-for-service private sector. The public sector is mainly governed by the Ministry of Health, which provides highly subsidised healthcare through hospital and clinic services. Besides that, there are several public healthcare facilities governed by the Ministry of Higher Education (university hospitals and clinics) and the Ministry of Defence (army hospitals and clinics). The private sector complements the public sector by providing health services for patients who opt to pay through out-of-pocket, private health insurance , or employers’ contributions (Malaysia National Health Accounts Section, 2021).

### Data source

The data used in this study were obtained from the IQVIA National Sales Audit Database, provided by the Pharmaceutical Services Programme, Ministry of Health (MOH). The database encompasses aggregated procurement and sales data of drug products in the public and private sectors. The public sector includes hospitals and clinics operated by the MOH, as well as data from government health facilities operated by other ministries; the private sector includes private hospitals, general practitioner clinics, community pharmacies, and other private organisations. Detailed information on this data are described elsewhere (Ministry of Health, 2024). Data on population size were obtained from the Department of Statistics Malaysia, based on the yearly population estimates of Malaysia (Department of Statistics Malaysia, 2025b).

### Antibiotics

All antibiotics for systemic use (WHO Anatomical Therapeutic Chemical (ATC) code: J01) were identified and grouped by antibiotic classes (World Health Organization, 2023). A total of seven antibiotic classes (carbapenems, cephalosporins, fluoroquinolones, macrolides, penicillins, tetracyclines, and others) and 57 antibiotic molecules were included in this study. Antibiotics grouped in the ‘others’ class include aminoglycosides, amphenicols, glycopeptides, glycylcyclines, lincosamides, lipopeptides, oxazolidinones, polymyxins, steroid antibacterials, sulfonamide-trimethoprim-combinations, and trimethoprim-derivatives. Data were available in unit quantity and unit price. Unit quantity and total amount of antibiotics were converted into defined daily dose (DDD) according to the WHO ATC/DDD index (World Health Organization, 2023). Unit price of each antibiotic was used to calculate cost expenditure.

### Outcome measures

Antibiotic utilisation rates were reported as DDD per 1000 inhabitants per day, using the Malaysian population of each corresponding year (Equation (1))

(1) $$\mathrm{DDD}\;\mathrm{per}\;1000\;\mathrm{inhabitants}\;\mathrm{per}\;\mathrm{day}=\;\frac{\mathrm{total}\;\mathrm{dose}\;\mathrm{of}\;\;\mathrm{drug},\;\;\mathrm{by}\;\mathrm{ATC}\;\mathrm{code}}{\mathrm{DDD}\;\times \mathrm{population}\;\times \mathrm{number}\;\mathrm{of}\;\;\mathrm{days}\;}\;\times 1000$$

Cost expenditure of antibiotic was measured by Malaysian Ringgit (MYR) and estimated using total cost for all units of antibiotics divided by population estimates (MYR per 1000 inhabitants). Antibiotics were classified into the ‘Access, Watch, Reserve’ categories according to the WHO AWaRe framework (Web Annex C, 2023), and the utilisation rate for each group was presented as a proportion (the DDDs of each group divided by total DDDs). The Access-to-Watch Index (AWI) was calculated by dividing the utilisation rate of Access antibiotics by the utilisation rate of Watch antibiotics. The Compound Annual Growth Rate (CAGR) (Equation (2)) was calculated to derive a comparable metric of antibiotic utilisation and expenditure across time (European Centre for Disease Prevention and Control (ECDC), 2014).

(2) $$\mathrm{CAGR}=\;{\left(\frac{C_{2023}}{C_{2019}\;}\right)}^{\frac{1}{4}}-1$$

C_2023_: Total antibiotic utilisation for the year 2023 (expressed in DDD per 1000 inhabitants per day).
C_2019_: Total antibiotic utilisation for the year 2019 (expressed in DDD per 1000 inhabitants per day).

### Statistical analysis

Descriptive analysis was used to present annual antibiotic utilisation from 2019 to 2023. Analysis was stratified by (1) antibiotic classes, (2) healthcare setting (primary care versus hospitals), (3) sectors (public versus private), and (4) route of administration (parenteral versus oral). Primary care includes data from the following facilities: public sector – public health clinics; private sector – general practitioner clinics and community pharmacies. Antibiotic expenditure was calculated by multiplying the average unit price by the sales volume data. This unit value should therefore be interpreted as a surrogate indicator of drug price that may not necessarily be the price paid by consumers. Expenditure data are presented in nominal and real terms, adjusted using the consumer price index with 2019 as the base year (Department of Statistics Malaysia, 2025a). Sensitivity analysis to assess clinical use estimates and minimise misclassification bias was conducted by including only data from public clinics and private clinics for comparison at the primary care level. All data processing and analysis was conducted using R software, version 4.1.0 (R Core Team, 2021).

## Results

**Figure 1.**
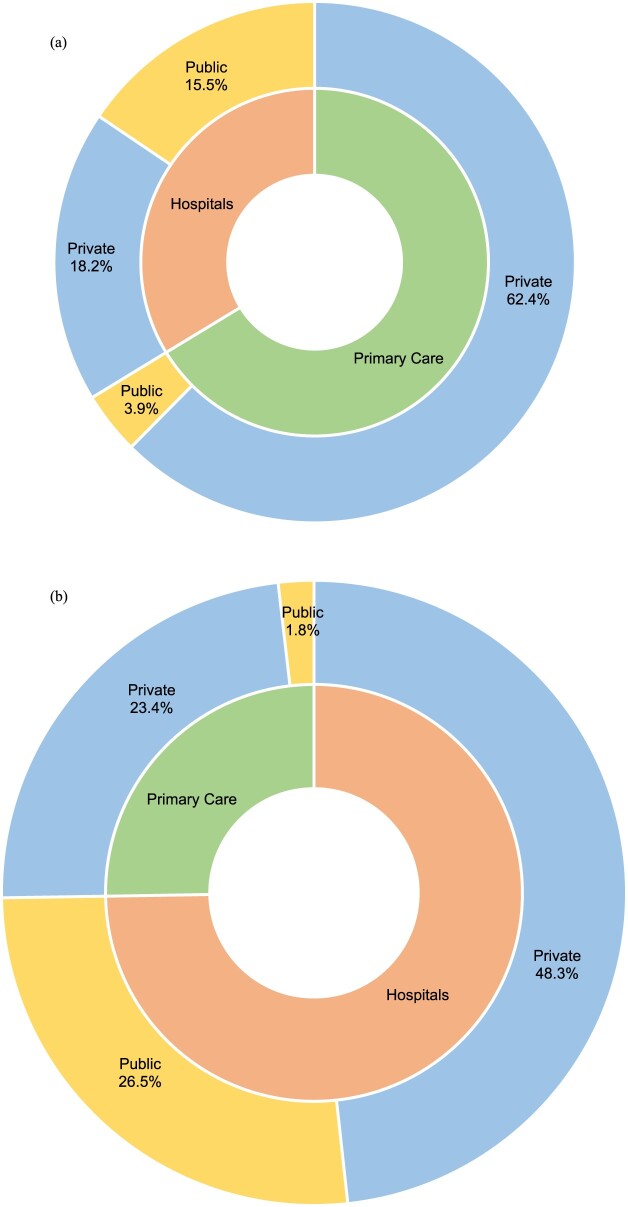
Proportion of (a) antibiotic utilisation (DDD per 1000 inhabitants per day), and (b) antibiotic expenditure (MYR) by level of care and sectors.

Figure 1 describes the share of antibiotics utilisation across healthcare settings and sector for 2019–2023. Primary care constituted 66.3% of the total antibiotic DDD per 1000 inhabitants per day, of which the majority belonged to the private sector. In comparison, the utilisation in public and private hospitals did not show such disparity (public hospitals 15.5%, private hospitals 18.2% of all DDD) (Figure 1(a)). Despite accounting for two-thirds of all antibiotic DDD per 1000 inhabitants per day, primary care accounted for only 25.3% of all expenditure on antibiotics in 2019–2023 (Figure 1(b)).

**Figure 2.**
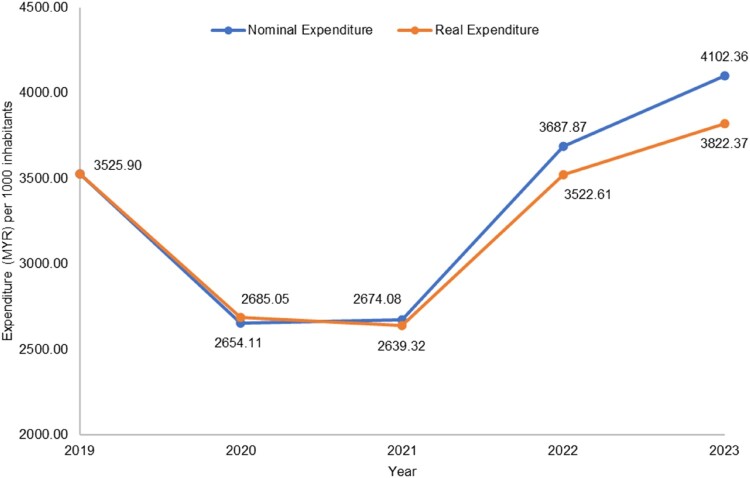
Total annual expenditure (MYR) per 1000 inhabitants in real and nominal terms, 2019–2023.

Total usage of antibiotics at the national level was 10.09 DDDs per 1000 inhabitants per day in 2019, decreasing slightly to 9.97 DDD in 2023, with a CAGR of −0.3%. The annual antibiotic utilisation rate from 2019 to 2023 by sector and care setting are shown in Table 1. In primary care, the antibiotic utilisation rate in both public and private sectors showed a fairly stable trend from 2019 to 2023. For hospitals, the antibiotic DDD decreased during 2020–2021, before picking up again in the following years. Overall, total annual nominal antibiotic expenditure increased by MYR296.47 per 1000 inhabitants over the study period from MYR3525.90 per 1000 inhabitants in 2019 to MYR4102.36 per 1000 inhabitants in 2023 (Table 1), with an average CAGR of 3.1%. In real terms, CAGR was lower at 1.6% where total annual real expenditure increased by RM296.47 per 1000 inhabitants in 2023 (Figure 2). While expenditure rose in the private sector (CAGR 4.9% in private primary care and 2.6% in private hospitals), the reverse was observed in the public sector (CAGR −5.9% in public primary care and −3.0% in public hospitals).

**Table 1.** Antibiotic (a) utilisation in DDD per 1000 inhabitants per day and (b) expenditure in MYR per 1000 inhabitants from 2019 to 2023, by sector and level of care.

(a)								
Level of Care	Sector	2019	2020	2021	2022	2023	CAGR	*p*-value
Total		10.09	9.13	8.73	9.75	9.97	−0.25%	0.8688
Primary Care	Public	0.37	0.37	0.37	0.37	0.36	−0.52%	0.1817
	Private	5.99	5.98	5.97	5.97	5.84	−0.52%	0.1118
Hospitals	Public	1.70	1.41	1.13	1.59	1.58	−1.44%	0.6312
	Private	2.03	1.37	1.26	1.82	2.18	1.51%	0.9457

(b)								
Level of Care	Sector	2019	2020	2021	2022	2023	CAGR	*p*-value
Total		3525.90	2654.11	2674.08	3687.87	4102.36	3.07%	0.3493
Primary Care	Public	90.17	49.20	37.87	55.37	71.41	−4.56%	0.6937
	Private	830.51	539.35	480.59	927.05	1134.40	6.43%	0.3081
Hospitals	Public	940.99	816.14	775.07	991.21	868.36	−1.59%	0.9322
	Private	1664.23	1249.42	1380.55	1714.24	2028.18	4.03%	0.2656

CAGR: Compound Annual Growth Rate

*p*-value was calculated using unpaired Student’s t-test to determine the statistical significance of observed trends.

**Figure 3.**
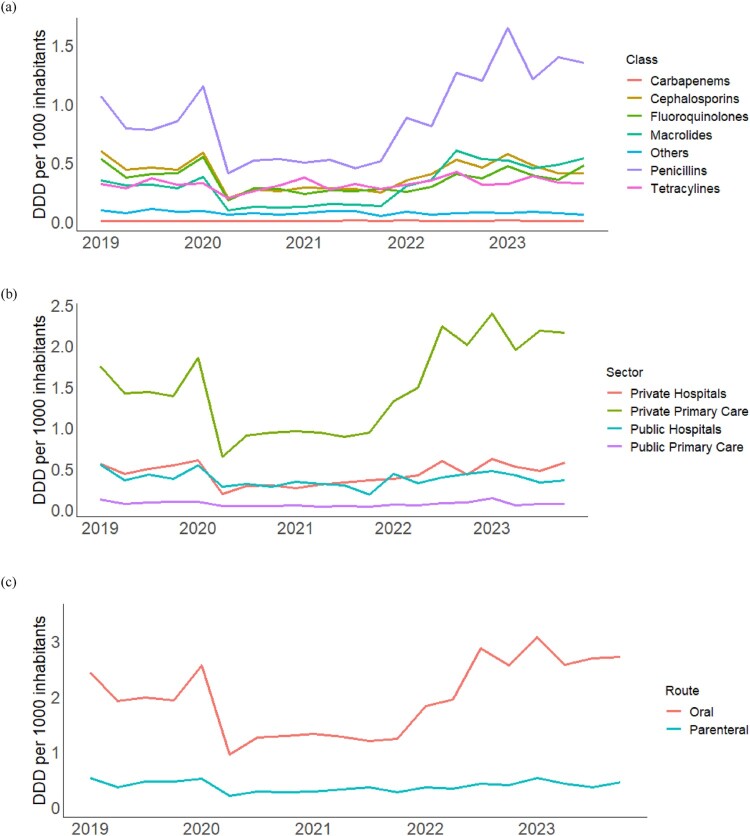
Quarterly Defined Daily Dose per 1000 inhabitants by (a) antibiotic classes, (b) level of care and sectors, and (c) route of administration.

Penicillins were the most commonly used antibiotics, followed by cephalosporins, macrolides, and fluoroquinolones as the classes of antibiotics with the highest utilisation. All classes of antibiotics had a drop in DDD during 2020–2021, but showed an increasing trend in the following years (Figure 3(a)). Private primary care had the highest proportion of antibiotics utilisation throughout the data period, where the overall penicillin utilisation mirrored its pattern of utilisation. While antibiotic utilisation in private and public hospitals was similar initially, antibiotic utilisation in private hospitals has been consistently higher from 2022 onwards (Figure 3(b)). Figure 3(c) shows overall parenteral and oral antibiotic utilisation between 2019 and 2023, where a much greater proportion of parenteral antibiotics was used in a hospital setting (46.6%) vs primary care (0.6%). During this period, parenteral antibiotics utilisation remained fairly stable while oral antibiotics utilisation was lower during 2020–2021.

**Figure 4.**
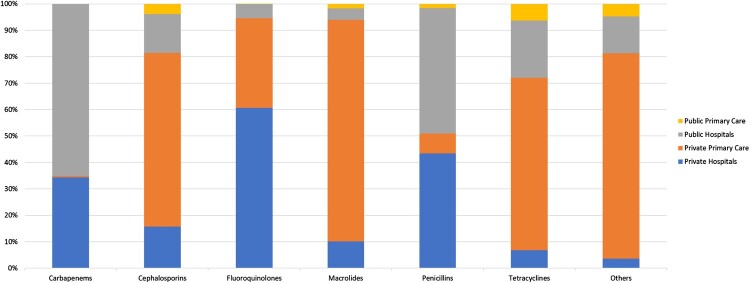
Antibiotic class utilisation stratified by healthcare setting from 2019–2023.

Amoxycillin in combination with clavulanic acid was among the top ten antibiotics used in all sectors by DDD and expenditure (Table 2). The top three antibiotics used in public primary care, private primary care, and public hospitals consist of antibiotics of penicillins, cephalosporins, and tetracyclines classes. In contrast, the top two antibiotics used in private hospitals were of fluoroquinolones class, followed by a cephalosporin. The majority of macrolides (83.9% of total DDD), tetracyclines (77.7%), and cephalosporins (65.8%) were used in private primary care, while 60.6% of fluoroquinolones were used in private hospitals and 65.2% of carbapenems were used in public hospitals (Figure 4).

**Table 2.** Proportion of top 10 antibiotics by utilisation (DDD per 1000 inhabitants per day) and expenditure (MYR) in (a) primary care and (b) hospitals.

(a)							
Primary Care							
Public				Private			
Utilisation (DDD)		Expenditure (MYR)		Utilisation (DDD)		Expenditure (MYR)	
Drug	Percentage (%)	Drug	Percentage (%)	Drug	Percentage (%)	Drug	Percentage (%)
cloxacillin	29.0%	cloxacillin	27.6%	amoxicillin + clavulanic acid	19.3%	amoxicillin + clavulanic acid	36.4%
amoxicillin	19.8%	amoxicillin + clavulanic acid	22.9%	doxycycline	16.2%	azithromycin	13.5%
cefalexin	18.8%	cefalexin	12.0%	amoxicillin	13.4%	cefuroxime	11.6%
doxycycline	16.4%	amoxicillin	11.6%	cefuroxime	11.4%	amoxicillin	8.7%
amoxicillin + clavulanic acid	4.5%	phenoxymethylpenicillin	8.6%	azithromycin	9.2%	clarithromycin	4.4%
erythromycin	3.7%	erythromycin	3.6%	cefalexin	8.9%	ampicillin + sulbactam	3.9%
phenoxymethylpenicillin	2.6%	cefuroxime	2.5%	ciprofloxacin	6.5%	cefalexin	3.7%
azithromycin	2.0%	ampicillin + enzyme inhibitor	2.4%	erythromycin	3.9%	erythromycin	3.0%
sulfamethoxazole + trimethoprim	1.3%	doxycycline	1.9%	clarithromycin	3.7%	ciprofloxacin	2.8%
cefuroxime	1.0%	benzylpenicillin	1.9%	cloxacillin	3.1%	cloxacillin	2.2%

(b)							
Hospitals							
Public				Private			
Utilisation (DDD)		Expenditure (MYR)		Utilisation (DDD)		Expenditure (MYR)	
Drug	Percentage (%)	Drug	Percentage (%)	Drug	Percentage (%)	Drug	Percentage (%)
amoxicillin + clavulanic acid	18.7%	amoxicillin + clavulanic acid	21.5%	moxifloxacin	26.9%	amoxicillin + clavulanic acid	21.5%
doxycycline	11.9%	cefuroxime	17.0%	ciprofloxacin	16.8%	cefuroxime	17.0%
amoxicillin	11.8%	ceftriaxone	9.9%	cefuroxime	12.9%	ceftriaxone	9.9%
cloxacillin	10.4%	cloxacillin	7.0%	amoxicillin + clavulanic acid	8.1%	cloxacillin	7.0%
cefuroxime	9.5%	meropenem	5.0%	levofloxacin	7.8%	meropenem	5.0%
linezolid	5.6%	ceftazidime	4.8%	linezolid	6.2%	ceftazidime	4.8%
ampicillin + enzyme inhibitor	3.8%	ampicillin + enzyme inhibitor	3.8%	azithromycin	3.7%	ampicillin + sulbactam	3.8%
ceftriaxone	3.6%	vancomycin	3.4%	clarithromycin	2.9%	vancomycin	3.4%
levofloxacin	3.5%	phenoxymethylpenicillin	2.6%	doxycycline	2.5%	phenoxymethylpenicillin	2.6%
cefalexin	2.1%	colistin	2.4%	ampicillin + enzyme inhibitor	2.5%	colistin	2.4%

**Figure 5.**
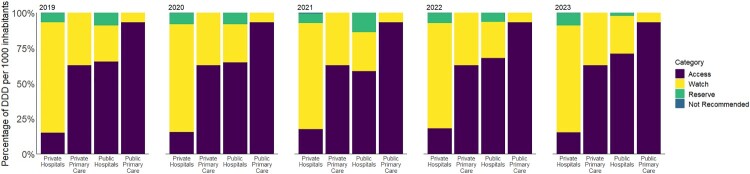
Annual proportion of antibiotic utilisation by AWaRe categories, sectors, and level of care.

When grouped by AWaRe categories, Access category antibiotics accounted for 56.0% of total antibiotic usage during 2019–2023. Within the primary care setting, 64.5% of total antibiotic utilisation was from the Access category (Figure 5). On the other hand, Access category antibiotics comprised only 39.2% of total hospital antibiotic utilisation. While public primary care consistently used more than 90% of Access category antibiotics, private primary care had lower usage of Access category antibiotics at 62.8% on average. Similarly, utilisation of Access category antibiotics was higher in public hospitals, ranging between 58.6% and 71.2% (with an average of 66.1%), while only 14.8–18.1% of antibiotic utilisation in private hospitals was Access category antibiotics. Between 2019 and 2023, the use of Reserve category antibiotics showed a fourfold decrease (from 8.8% to 2.1%) in the public sector, but increased from 6.6% to 8.9% in the private sector. The detailed breakdowns of the proportion of antibiotics by AWaRe categories across the healthcare settings throughout the study period are provided in Supplemental Table S1. The overall AWI was similar in 2019 (AWI = 1.29) and 2022–2023 (AWI = 1.28) but was slightly elevated during 2020–2021 (AWI = 1.42). Private hospitals had higher rates of Watch antibiotic usage, which resulted in AWI of less than 1 (AWI = 0.21) as compared to public primary care (AWI = 13.9), public hospitals (AWI = 2.50), and private primary care (AWI = 1.69).

Within the primary care level, community pharmacies accounted for approximately 20% of antibiotic use and expenditure at the private sector. When only data from public clinics and private clinics were included for comparison at primary care, similar patterns were observed (Supplemental Table S2).

## Discussion

This study provides a comprehensive analysis of the landscape of national antibiotic utilisation and expenditure in Malaysia. While previous studies have reported antibiotic utilisation from single or multicenter studies or sector-specific analyses, this is the first comprehensive study on national antibiotic utilisation in recent years in Malaysia, with detailed analysis and breakdown according to sectors (public and private), levels of healthcare (primary care and hospitals), as well as the AWaRe classification of antibiotics.

Overall, our study found that antibiotic utilisation rates were fairly consistent during the study period, except during 2020–2021 where a downward trend was observed particularly in the hospital setting. This trend reflected the COVID-19 pandemic period where changes in the antibiotic utilisation rate was similarly reported in other studies during the peak of the pandemic (Högberg et al., 2021; Meschiari et al., 2022; Mohamad et al., 2022; Nandi et al., 2023). Reductions in antibiotic usage during the COVID-19 pandemic could be attributed to several factors. Non-pharmaceutical interventions for COVID-19 such as mask-wearing, social distancing, and restrictions on travel and gatherings have resulted in reductions in the incidence of infectious diseases which led to reductions in antibiotic usage (Ullrich et al., 2021; Zhang et al., 2023). In addition, reduced access to healthcare and a decrease in the number of consultations and elective procedures due to lockdown may have also resulted in the decrease in antibiotic utilisation during this period (Amaran et al., 2021; Mehta et al., 2022).

In this study, we observed distinguishable patterns of the antibiotic consumption between the public and private healthcare sectors in Malaysia. Approximately two-thirds of total antibiotics utilisation was utilised in primary care, with private facilities accounting for the majority. Other studies have previously reported that primary care or community healthcare accounted for more than 70% of antibiotic consumption (Duffy et al., 2018). Our findings showed a slightly lower proportion, but the differences could be explained by the different healthcare setups between countries. Although primary care clinics function as gatekeepers, patients in Malaysia may still directly access secondary or tertiary private healthcare facilities without a physician referral (Ong et al., 2022). In addition, the underlying burden of infection and available healthcare resources may have contributed to a higher proportion of cases being managed in hospitals. This is lower than that reported in the United Kingdom (70%) (UK Health Security Agency (UKHSA), 2025) and New Zealand (85–95%) (Duffy et al., 2018). This could be due to a higher burden of infection and a lower threshold for admission in the local setting. In the public sector, hospital antibiotic consumption was higher than in primary care, whereas in the private sector, the reverse was observed. This difference may be attributed to the types of cases managed in each setting, as private primary care tends to treat more acute conditions, whereas public primary care primarily manages chronic diseases (Sivasampu et al., 2016). This is consistent with study findings where parenteral antibiotics comprised much more of total antibiotic utilisation in the hospital setting compared to primary care.

The AWaRe antibiotic classification provides a useful framework for quantitative assessment of antibiotic use patterns. Our findings show that in 2019–2023, the proportion of Access antibiotic use met the (MyAP-AMR) 2022–2026 target of at least 60% of total antibiotic consumption being from the Access category (Ministry of Health Malaysia, 2022). Restricted formulary options in the public sector, especially in primary care clinics, may have contributed to more prudent prescribing of antibiotics, which led to a consistently high proportion of Access antibiotic use at more than 90% in public clinics and an improvement in public hospitals over the years. Since 2014, the AMS protocol was adopted in healthcare facilities across Malaysia to formalise the formation of AMS in the healthcare system (Ministry of Health, 2022). Despite the nationwide implementation and advocacy involving both public and private healthcare facilities, the implementation was more rigorous in the public sector (both hospitals and clinics), which may have also contributed to more standardised antibiotic prescribing practices in the public sector (Ministry of Health, 2022; Ministry of Health and Ministry of Agriculture & Agro-Based Industry Malaysia, 2017). Nevertheless, this finding highlights the need for further enhancement in stewardship efforts for a successful implementation of policies to improve antibiotic usage in the country. Further initiatives will need to be implemented in order to achieve the latest WHO target of Access category antibiotics comprising 70% of total antibiotic consumption by 2030 (News and Media Unit United Nations Environment Programme, 2024).

The higher proportion of Watch and Reserve antibiotics in the hospitals reflects the availability of higher-end and restricted antibiotics in the secondary and tertiary care. Notably, our findings showed that Watch antibiotics accounted for 70% of antibiotics use in private hospitals compared to 26% in public hospitals. Given that private healthcare facilities in Malaysia are accessible by fee-for-service model, patients with infectious disease may choose to seek care at private hospitals for faster service. At the same time, private hospitals have less restriction on antibiotics selection compared to cost-containment strategy employed in public hospitals, which could contribute to higher proportion of Watch and Reserve antibiotics use. However, our findings alone do not reflect the appropriateness of antibiotics use as we do not have information on diagnoses and indications. Besides, our study observed that over 90% of fluoroquinolone utilisation was contributed by the private sector, as well as high utilisation of third-generation cephalosporins. The judicious use of fluoroquinolones is important as the resistance to this class of antibiotics has become widespread in a number of pathogens, largely driven by the overuse and misuse of fluoroquinolones, such that fluoroquinolone resistance in *E. coli* has been documented to be as high as 84% (Manik et al., 2025). The widespread use of third generation cephalosporins is known to be a major driver of ESBLs that could further confer resistance to broad spectrum cephalosporins and monobactams (Sirot, 1995). A meta-analysis by Sulis et al. indicated a strong association between the use of third-generation cephalosporins and colonisation/infection with ESBL *Enterobacterales* while prior exposure to fluoroquinolones was associated with methicillin resistant *Staphylococcus aureus* (Sulis et al., 2022). This trend highlights the need for targeted stewardship efforts to limit the inappropriate use of higher-risk antibiotics, especially in private healthcare settings.

Despite almost equal distribution of patients who sought outpatient healthcare services in the public (48.9%) and private (51.1%) sectors reported in the Malaysia National Health and Morbidity Survey 2023 (Institute for Public Health, 2024), antibiotic utilisation in the private sector was approximately four times higher than in the public sector. Additionally, although hospital admissions were rising from 2019 to 2023, we observed contrasting trends in antibiotic utilisation: its usage increased in private hospitals, but it declined in public hospitals. This relatively higher usage of antibiotics in the private sector may be explained by the previously conducted economic analysis of healthcare expenditures in LMICs by the WHO, which highlighted a correlation between out-of-pocket expenditure and overprescription of antibiotics due to supply-side incentives and less standardised quality assurance (Alsan et al., 2015). Previous studies have also demonstrated varying rates of antibiotic prescribing across different prescribers in private and public primary care, contributed to by the diverse challenges and drivers including prescribers’ knowledge and experience, patients’ requests and expectations, and adherence to current guidelines (Hassali et al., 2015; Rezal et al., 2015; Saleem, Moore, et al., 2025). When compared to other countries in the region, the average national DDD per 1000 inhabitants of 9.53 in Malaysia was higher than that of Indonesia (5.3 DDD) and the Philippines (5.0 DDD), but lower than that of Thailand (12.4 DDD) and Vietnam (30.0 DDD), using data from the same database (Browne et al., 2021). A direct comparison of the national antibiotic utilisation rates, however, is challenging due to the lack of studies utilising similar data sources.

The rising use and cost of antibiotics have major public health and economic implications, driven by increasing resistance that leads to longer hospital stays and costlier treatments (Dadgostar, 2019). In Malaysia, systemic antibacterials ranked among the top in medication expenditure from 2019–2022 (Ministry of Health, 2024). Strengthening antimicrobial stewardship (AMS) programmes – especially in private hospitals – alongside public education (Calò et al., 2021), the AWaRe framework (Mendelson et al., 2024), enhanced surveillance, and policy incentives can improve prescribing practices, reduce unnecessary use, and control costs (Ajulo & Awosile, 2024; Bertagnolio et al., 2023). Expansion of MyAP-AMR for more active involvement of the private sector can encourage more judicious use of antibiotics. Collaborative efforts between public and private sectors are essential to ensure consistent, cost-effective, and rational antibiotic use while mitigating the risks of antimicrobial resistance (Fernow et al., 2025).

This study's key strengths include the use of comprehensive national data, allowing for a robust analysis of antibiotic utilisation and expenditure trends. By analysing real expenditure, changes in antibiotic utilisation are isolated from broader economic inflation. However, some limitations must be acknowledged. This study analysed population-level data on antibiotics purchased by and sold to healthcare facilities, which may not represent the true antibiotic intake. Data on individual patient characteristics and clinical indications were not available, limiting the ability to assess appropriateness of antibiotic use. Additionally, the study did not assess the impact of antibiotic utilisation on antimicrobial resistance and clinical outcomes. Detailed information on geographic region and brand name was also not available in the current dataset, which would otherwise allow greater depth in the analysis of utilisation by rural vs urban, as well as brand name vs generic. Lastly, as real expenditure was calculated using the consumer price index, we were unable to adjust using the pharmaceutical price index, and as such may not reflect healthcare and drug-specific inflation.

Future studies should explore the relationship between antibiotic utilisation, resistance patterns, and patient outcomes. There is also a crucial need to assess the appropriateness of antibiotic prescribing, especially for the Watch and Reserve antibiotics. Patient-level data, if available, would allow for a more detailed analysis of prescribing appropriateness and treatment effectiveness. Longitudinal studies assessing the long-term cost-effectiveness of antibiotic stewardship interventions are important as well. Further research should also look into examining the impact of public health policies on antibiotic use and expenditure, particularly in the context of emerging infectious diseases.

## Conclusion

Our findings show that primary care contributes to two-thirds of the total antibiotic utilisation in Malaysia from 2019 to 2023. Antibiotic utilisation in the private sector was higher than the public sector for both primary care and hospitals. Overall, the Access category antibiotic utilisation falls short of the 60% target set by the WHO during the study period. Lower rates of Access category antibiotics were observed in the private sector, particularly private hospitals. These findings underscore the need for and can contribute to guiding future robust and targeted antibiotic stewardship programmes, evidence-based prescribing guidelines, and effective healthcare policies to optimise antibiotic utilisation and, at the same time, mitigate the impacts of antibiotic resistance in Malaysia.

## Ethics approval

This study was registered in the National Medical Research Register and approved by the Medical Research and Ethics Committee (MREC), Ministry of Health Malaysia (NMRR ID 23-00234). Informed consent was waived by MREC as only aggregated sales data were used, with no access to patient-level data or identifiable information.

## Supplementary Material

NAU_List of Supplementary Tables_v2.docx

## Data Availability

The datasets used and/or analysed during the study are available from the corresponding author on reasonable request.
