# Supplementary material for: Landscape of national antibiotic utilisation in Malaysia: an analysis of national pharmaceutical sales data from 2019 to 2023
Source: J Pharm Policy Pract. 2026 May 21;19(1):2668477. doi: 10.1080/20523211.2026.2668477 (PMC13195718; doi:10.1080/20523211.2026.2668477)
Supplement: NAU_List of Supplementary Tables_v2.docx [file JPPP_A_2668477_SM9798.docx]

# Title

Landscape of national antibiotic utilisation in Malaysia: an analysis of national pharmaceutical sales data from 2019 to 2023

# List of Supplementary Tables

Supplementary Table S1: Annual utilisation in DDD per 1000 inhabitants per day and proportion of antibiotic utilisation by AWaRe categories, sectors, and level of care.

Supplementary Table S2: Antibiotic (a) utilisation in DDD per 1000 inhabitants per day and (b) expenditure in MYR per 1000 inhabitants from 2019 to 2023, for primary care clinics only.

**Supplementary Table S1: Annual utilisation in DDD per 1000 inhabitants per day (DID) and proportion of antibiotic utilisation by AWaRe categories, sectors, and level of care.**

| **Healthcare Setting & Year** | **Access** | | **Watch** | | **Reserve** | | **Not Recommended** | | **Total**  **DID** |
| --- | --- | --- | --- | --- | --- | --- | --- | --- | --- |
|  | **DID** | **Proportion** | **DID** | **Proportion** | **DID** | **Proportion** | **DID** | **Proportion** |  |
| **Private Hospitals** | **1.40** | **16.15%** | **6.59** | **76.16%** | **0.65** | **7.46%** | **0.02** | **0.23%** | **8.66** |
| 2019 | 0.30 | 14.84% | 1.59 | 78.51% | 0.13 | 6.42% | 0.00 | 0.23% | 2.03 |
| 2020 | 0.21 | 15.62% | 1.05 | 76.26% | 0.11 | 7.86% | 0.00 | 0.26% | 1.37 |
| 2021 | 0.22 | 17.45% | 0.95 | 75.27% | 0.09 | 6.96% | 0.00 | 0.32% | 1.26 |
| 2022 | 0.33 | 18.08% | 1.36 | 74.64% | 0.13 | 7.11% | 0.00 | 0.17% | 1.82 |
| 2023 | 0.34 | 15.34% | 1.65 | 75.71% | 0.19 | 8.75% | 0.00 | 0.20% | 2.18 |
| **Private Primary Care** | **18.68** | **62.76%** | **11.08** | **37.22%** | **0.00** | **0.01%** | **0.00** | **0.00%** | **29.76** |
| 2019 | 3.76 | 62.76% | 2.23 | 37.22% | 0.00 | 0.01% | 0.00 | 0.00% | 5.99 |
| 2020 | 3.76 | 62.76% | 2.23 | 37.22% | 0.00 | 0.01% | 0.00 | 0.00% | 5.98 |
| 2021 | 3.75 | 62.76% | 2.22 | 37.22% | 0.00 | 0.01% | 0.00 | 0.00% | 5.97 |
| 2022 | 3.75 | 62.76% | 2.22 | 37.22% | 0.00 | 0.01% | 0.00 | 0.00% | 5.97 |
| 2023 | 3.67 | 62.76% | 2.17 | 37.22% | 0.00 | 0.01% | 0.00 | 0.00% | 5.84 |
| **Public Hospitals** | **4.89** | **66.09%** | **1.96** | **26.45%** | **0.55** | **7.45%** | **0.00** | **0.00%** | **7.40** |
| 2019 | 1.11 | 65.48% | 0.44 | 25.72% | 0.15 | 8.80% | 0.00 | 0.00% | 1.70 |
| 2020 | 0.91 | 64.99% | 0.38 | 26.99% | 0.11 | 8.02% | 0.00 | 0.00% | 1.41 |
| 2021 | 0.66 | 58.56% | 0.31 | 27.78% | 0.15 | 13.66% | 0.00 | 0.00% | 1.13 |
| 2022 | 1.08 | 68.06% | 0.41 | 25.55% | 0.10 | 6.39% | 0.00 | 0.00% | 1.59 |
| 2023 | 1.12 | 71.16% | 0.42 | 26.72% | 0.03 | 2.13% | 0.00 | 0.00% | 1.58 |
| **Public Primary Care** | **1.72** | **93.28%** | **0.12** | **6.71%** | **0.00** | **0.00%** | **0.00** | **0.00%** | **1.84** |
| 2019 | 0.35 | 93.28% | 0.02 | 6.71% | 0.00 | 0.00% | 0.00 | 0.00% | 0.37 |
| 2020 | 0.35 | 93.28% | 0.02 | 6.71% | 0.00 | 0.00% | 0.00 | 0.00% | 0.37 |
| 2021 | 0.34 | 93.28% | 0.02 | 6.71% | 0.00 | 0.00% | 0.00 | 0.00% | 0.37 |
| 2022 | 0.34 | 93.28% | 0.02 | 6.71% | 0.00 | 0.00% | 0.00 | 0.00% | 0.37 |
| 2023 | 0.34 | 93.28% | 0.02 | 6.71% | 0.00 | 0.00% | 0.00 | 0.00% | 0.36 |
| **Overall** | **26.69** | **56.00%** | **19.75** | **41.44%** | **1.20** | **2.52%** | **0.02** | **0.04%** | **47.66** |
| 2019 | 5.52 | 54.72% | 4.28 | 42.45% | 0.28 | 2.78% | 0.00 | 0.05% | 10.09 |
| 2020 | 5.23 | 57.26% | 3.68 | 40.27% | 0.22 | 2.42% | 0.00 | 0.04% | 9.13 |
| 2021 | 4.97 | 56.99% | 3.51 | 40.19% | 0.24 | 2.78% | 0.00 | 0.05% | 8.73 |
| 2022 | 5.50 | 56.44% | 4.01 | 41.15% | 0.23 | 2.37% | 0.00 | 0.03% | 9.75 |
| 2023 | 5.46 | 54.81% | 4.27 | 42.88% | 0.23 | 2.26% | 0.00 | 0.04% | 9.97 |

**Supplementary Table S2: Antibiotic (a) utilisation in DDD per 1000 inhabitants per day (DID) and (b) expenditure in MYR per 1000 inhabitants from 2019 to 2023, for primary care clinics only.**

(a)

| **Sector** | **2019** | **2020** | **2021** | **2022** | **2023** | **CAGR** | **p-value** |
| --- | --- | --- | --- | --- | --- | --- | --- |
| Public | 0.37 | 0.37 | 0.37 | 0.37 | 0.36 | -0.52% | 0.1817 |
| Private | 4.92 | 4.91 | 4.90 | 4.90 | 4.79 | -0.52% | 0.1022 |

(b)

| **Sector** | **2019** | **2020** | **2021** | **2022** | **2023** | **CAGR** | **p-value** |
| --- | --- | --- | --- | --- | --- | --- | --- |
| Public | 90.17 | 49.20 | 37.87 | 55.37 | 71.41 | -4.56% | 0.6937 |
| Private | 696.14 | 444.06 | 384.32 | 759.24 | 945.52 | 6.31% | 0.3305 |

CAGR: Compound Annual Growth Rate

p-value was calculated using unpaired Student’s t-test
